# Predictive Markers of Early Cardiovascular Impairment and Insulin Resistance in Obese Pediatric Patients

**DOI:** 10.3390/diagnostics11040735

**Published:** 2021-04-20

**Authors:** Laura Mihaela Trandafir, Elena Cojocaru, Mihaela Moscalu, Maria Magdalena Leon Constantin, Ingrith Miron, Alexandra Mastaleru, Oana Teslariu, Madalina Elena Datcu, Silvia Fotea, Otilia Frăsinariu

**Affiliations:** 1 Department of Mother and Child, Grigore T. Popa University of Medicine and Pharmacy, 700115 Iasi, Romania; trandafirlaura@yahoo.com (L.M.T.); ingridmiron@hotmail.com (I.M.); oana.teslariu@gmail.com (O.T.); otiliafrasinariu@gmail.com (O.F.); 2 Department of Morphofunctional Sciences I, Grigore T. Popa University of Medicine and Pharmacy, 700115 Iasi, Romania; ellacojocaru@yahoo.com; 3 Department of Preventive Medicine and Interdisciplinarity, Grigore T. Popa University of Medicine and Pharmacy, 700115 Iasi, Romania; 4 Department of Medical Specialties I, Grigore T. Popa University of Medicine and Pharmacy, 700115 Iaşi, Romania; alexandra.mastaleru@gmail.com; 5 Department of Biomedical Sciences, Grigore T. Popa University of Medicine and Pharmacy, 700115 Iasi, Romania; madalina.dtc@gmail.com; 6 Department of Medical Specialties, Dunarea de Jos University, 800216 Galati, Romania; silvia_ghimpu@yahoo.com

**Keywords:** obese pediatric patients, cardiovascular, inflammation markers, insulin resistance markers, comorbidities

## Abstract

Background: The increased prevalence of obesity among children determined the rising number of its comorbidities in children and adults, too. This study aimed to evaluate certain markers of inflammation and insulin resistance in obese pediatric patients, identifying those who are more likely to develop further complications. Methods: We included 115 obese pediatric patients: 85 overweight and obese patients in the study group and 30 normal-weight patients in the control group. We calculated the body mass index (BMI) and we evaluated markers (biological, inflammatory) and the hormones profile. Results: Low-threshold inflammation was assessed by measuring interleukin 6 IL-6 and Intercellular Adhesion Molecules (ICAM). The analysis showed that IL-6 is significantly correlated with glucose (*p* = 0.001) and BMI value (*p* = 0.031). ICAM correlates significantly with triglycerides (*p* = 0.001), glucose (*p* = 0.044) and BMI percentile (*p* = 0.037). For pediatric obese patients, endotoxemia has been significantly correlated only with BMI percentile (*p* = 0.001). Plasma cortisol did not show significant correlations with total cholesterol, triglycerides, glucose or BMI percentile. The results indicated a significant predictive power of BMI percentile on inflammatory markers: IL-6 (AUC = 0.803, *p* < 0.001), ICAM (AUC = 0.806, *p* < 0.001) and endotoxemia (AUC = 0.762, *p* = 0.019). Additionally, BMI percentile has a significant predictive power for metabolic markers of insulin resistance (insulin value: AUC = 0.72, *p* < 0.001 and HOMA index: AUC = 0.68, *p* = 0.003). Conclusions: The study highlighted the importance of early markers of cardiovascular risk in obese pediatric patients represented by IL-6, ICAM, endotoxemia and their correlation with metabolic markers of insulin resistance represented by insulinemia, HOMA index and plasma cortisol. It can clearly be considered that the BMI percentile has significant predictive power for metabolic markers of insulin resistance.

## 1. Introduction

The new obesity epidemic encountered in children and adolescents brings along an increased risk of multiple comorbidities and mortality at a young age [1]. Many studies show that childhood obesity will affect a future adult’s health. The link between obesity and the increased risk of other diseases, including cardiovascular disease (CVD), type 2 diabetes mellitus (T2DM), neoplasia, is well established [2,3,4,5]. Insulin resistance (IR) and chronic inflammation have an essential role in the pathogenesis of obesity-associated comorbidities [6,7]. Early identification of signs of cardiovascular impairment and insulin resistance (IR) in obese children and adolescents is essential for the precocious establishment of lifestyle correction measures and ensuring long-term health. Thus, early management of childhood obesity aims not only to lower BMI but delays the onset of early CVD and T2DM [8,9]. Early CVD predictors include BMI, hypertension, dyslipidemia, IR and elevated circulating inflammatory molecules [10]. Inflammatory changes during the early stages of childhood obesity impair metabolic and cardiovascular health [11]. The adipose tissues release many inflammatory mediators which predispose to a pro-inflammatory state and oxidative stress.

Among the markers of chronic inflammation, interleukin 6 (IL-6) is an adipocytokine with a pro-inflammatory role and contributes to IR. Studies have shown that high levels of IL-6 are associated with metabolic or cardiac comorbidities in adulthood. Additionally, systemic, low-level elevations of gut-derived endotoxin has a role in metabolic changes in obesity [12]. Metabolic endotoxemia is related to systemic and local inflammation, and therefore, may contribute, at least in part, to cardio-metabolic disease risk associated with obesity [13].

One of the crucial aims of preventing the comorbid conditions associated with obesity is to detect children with higher cardiovascular risk at an early stage [14]. In this study, we evaluated the presence of early markers of cardiovascular risk represented by interleukine-6 (IL-6), Intercellular Adhesion Molecules (ICAM) and endotoxemia and their correlation with metabolic markers of IR represented by insulinemia, HOMA index and plasma cortisol.

## 2. Materials and Methods

### 2.1. Study Design

We conducted a prospective study on two groups of pediatric patients: the study group and the control group. The study group included 85 obese pediatric patients between 6 and 18 years old with obesity without associated pathologies followed in Children′s Hospital “Sfânta Maria” Iaşi between 1 January and 31 December 2019. The control group included 30 pediatric patients with normal BMI. The inclusion criteria were patients with newly diagnosed obesity who did not benefit from dietary and/or pharmacological treatment. The exclusion criteria were smoking, pregnancy, secondary and genetic causes of obesity, cardiovascular diseases in treatment and other chronic diseases, autoimmune diseases, hormonal abnormalities (thyroid diseases, polycystic ovary syndrome, secondary amenorrhea), or administration of any chronic therapy in the previous three months. Only children and adolescents who provided informed consent (including parental consent) were included in the study.

Out of the 133 children and adolescents with obesity included in the study, we excluded 24 children and adolescents with cardiovascular disease with dietary and pharmacological treatment, 6 with secondary amenorrhea and 18 children and adolescents with chronic diseases with dietary and pharmacologic treatment. Children and adolescents were not divided in two separate age-related groups because of a lack of untreated obese adolescents with a formal diagnosis made more than 3 years prior. We also found no statistically significant changes in the assessment of inflammatory markers in the two age groups (pre-pubertal and adolescent). Therefore, we performed statistical processing on the entire group of 85 children and adolescents.

### 2.2. Anthropometric and Biochemical Measurements

The followed anthropometric parameters were body weight, body height, waist circumference and BMI. The interpretation of BMI values was made according to the BMI percentile, applicable for age and sex, according to the CDC standards. Overweight is defined as a BMI at or above the 85th percentile and below the 95th percentile for children and teens of the same age and sex. Obesity is defined as a BMI at or above the 95th percentile for children and teens of the same age and sex. Depending on the BMI percentile adjusted for age and gender, patients were classified into two categories: obese (BMI percentiles between 95–99th) and severely obese (BMI percentiles > 99th). We defined visceral obesity by WC values above the 90th percentile. In all patients, the blood pressure (BP) value was determined, and the obtained values were evaluated according to the percentiles for age and sex. The blood pressure (BP) value was interpreted according to the National High Blood Pressure Education Program Working Group on High Blood Pressure in Children and Adolescents. Children hypertension was defined as systolic BP and/or diastolic BP > 95th percentile, adjusted for height, age and sex, with at least three separate measurements. Normally, high blood pressure was considered for values between the 90 and 95 percentile values (National Institute for Health, 2004). In our study, BP values ≥ 90th percentile were defined as “elevated BP” or vascular impairment.

Total lipid profiles, liver function tests, total protein, blood glucose and creatinine were measured. The reference standards were used to interpret the values of biological parameters. The cardiovascular risk parameters were defined by age and sex. Borderline cardiovascular risk was considered when the total cholesterol was between 180–199 mg/dL. Medium cardiovascular risk was defined as a cholesterol value between 200–249 mg/dL or an LDL-cholesterol (low density lipoprotein-cholesterol) value between 130–159 mg/dL according to SINUPE 2000. High cardiovascular risk was defined by a cholesterol value over 250 mg/dL or an LDL-cholesterol value over 160 mg/dL.

IL 6, ICAM, endotoxemia, insulinemia, plasma cortisol and HOMA-IR (Homeostasis model assessment) evaluated the inflammatory and metabolic status. We used enzyme-linked immunosorbent assay (ELISA) kits for the quantitative detection of human IL-6, sICAM-1 and cortisol. The ELISA kits are for research use only, not for diagnostic or therapeutic procedures. Cortisol was determined using an ELISA kit in human serum. The reference values for cortisol in serum were 60–230 ng/mL. For determining IL-6 and ICAM-1, we used BioVendor kits. The amount of IL-6 or ICAM-1 was determined extrapolating OD values against IL-6/ICAM-1 standard concentrations using a standard curve. The minimum detectable concentrations were 0.10 pg/mL for IL-6, 2.2 ng/mL for ICAM-1 and the interassay coefficient of variation was 7.0% for all kits. Endotoxin (ET) was determined using the Abbexa ELISA kit. The sensitivity is <0.005 EU/mL and the concentrations range of ET in serum 0.015 EU/mL–1.0 EU/mL using the Abbexa ET kit.

Informed consent was obtained from all participants and their parents in accordance with the Helsinki Declaration revised in 2013. The study was approved by the Ethical Committee of the Saint Mary Children Hospital (14055/19 June 2018).

### 2.3. Statistical Analysis

Statistical analysis of the data was performed using the STATA 16 software (StataCorp LLC, College Station, TX, USA). Continuous variables were presented as mean values and standard deviation or as median with lower and upper quartile (Q1; Q3). To compare the values of the continuous type parameters corresponding to the two groups of patients we applied the ANOVA test or Mann–Whitney U Test depending on the homogeneity of the data series (Levene’s test). Qualitative variables were compared based on Pearson Chi-square test results. To verify the correlations between the continuous type variables, we applied the Pearson univariate correlation test. Quantitative analysis of the contribution of each biochemical parameter and BMI in the modification of inflammatory markers and metabolic markers of insulin resistance was performed based on the coefficients of multiple linear regression. At the same time, their predictive power was estimated based on the receiver operating characteristic (ROC) curve and the AUC value (area under the ROC curve). In the statistical analysis, the reference threshold for the level of significance *p* was 0.05. A *p* value < 0.05 indicated with 95% confidence that there was statistical significance.

## 3. Results

### 3.1. Patient Characteristics

We included 115 children aged between 6 and 18 years old. There were no significant differences in the two study groups related to age and gender of the children. In the control group, the mean age was 13.4 ± 2.47 years and for the study group, the mean age was 12.1 ± 3.4 years. In Table 1, we present the main characteristics of the included patients. Regarding the lipid profile, we observed significant higher values of the triglycerides and lower levels of HDL cholesterol in the obese patients. We observed significant higher levels of HbA1c, insulinemia and HOMA IR index in obese patients. Moreover, inflammatory markers, IL-6, ICAM 1 and endotoxemia were significantly higher in obese patients versus the control group.

### 3.2. Correlations between the BMI Percentile and Biochemical Markers with Inflammatory Markers of Early Cardiovascular Risk (IL-6, Endotoxemia and ICAM 1)

Regarding the changes of the inflammatory markers of the obese patients included in the study, we identified significant correlations between them and BMI. Significant correlations were also identified between certain biochemical parameters and the level of the analyzed inflammatory markers. Thus, IL-6 correlates significantly with blood glucose (*r* = −0.334, *p* = 0.001) and BMI percentile (*r* = 0.252, *p* = 0.031) (Table 2). We also found a significant correlation between ICAM and serum triglycerides values (*r* = 0.253, *p* = 0.001), plasma glucose level (*r* = −0.145, *p* = 0.044) and with BMI (r = 0.302, p = 0.037). Additionally, in the context of obesity, the results indicated a significant correlation between endotoxemia and plasma glucose level (*r* = 0.346, *p* = 0.024) but also with BMI percentile (*r* = −0.255, *p* = 0.001) (Table 2).

**Figure 1:**
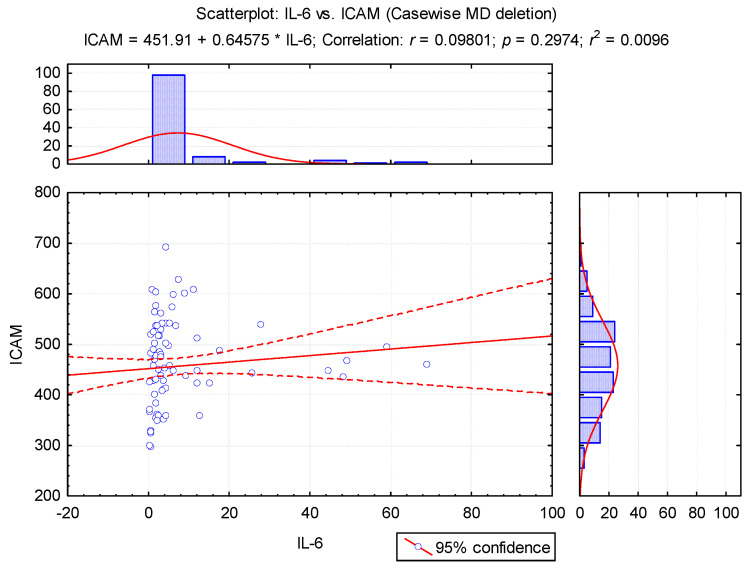
The regression line in the correlation of IL-6 and ICAM values.

Thus, we observed that IL-6 was significantly correlated with blood glucose and BMI percentile, these being significant predictive factors for cardiometabolic diseases. The link between low-grade inflammation, IR and endothelial dysfunction and obesity is currently being studied extensively. Low-grade inflammation was assessed in our study by measuring both IL-6 and ICAM. We did not find a positive correlation (*r* = 0.09, *p* = 0.297) between the values of IL-6 and ICAM in the analyzed groups (Figure 1).

**Figure 2:**
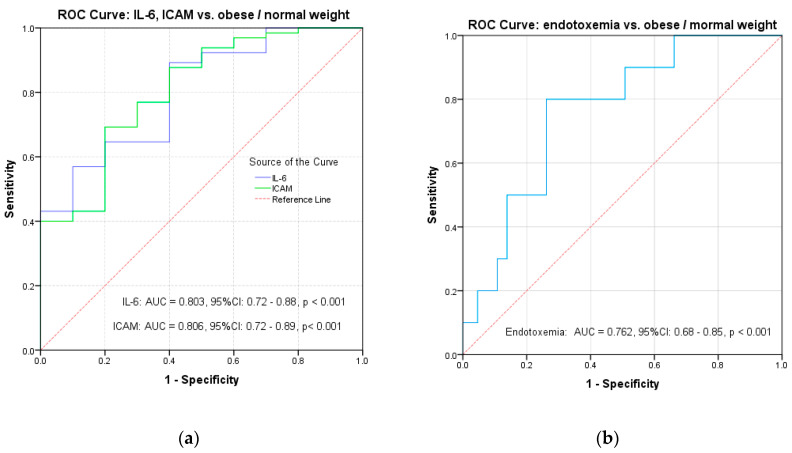
ROC curves for BMI vs. (**a**) IL-6 and ICAM and (**b**) endotoxemia.

**Figure 3:**
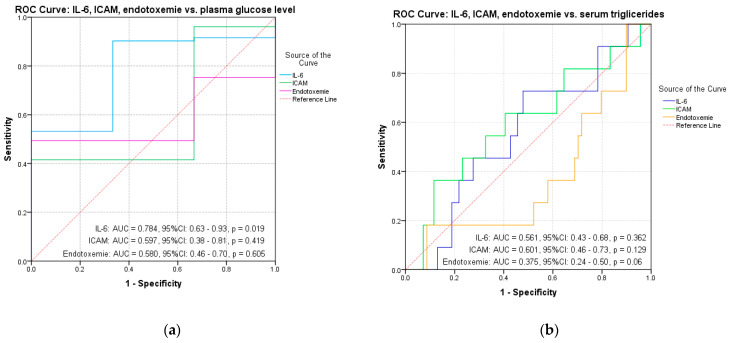
ROC curves for IL-6 and ICAM and endotoxemia vs. (**a**) plasma glucose level and (**b**) serum triglycerides.

Starting from the results of the univariate analysis, we made a multivariate analysis to evaluate the contribution of each biochemical parameter but also of BMI percentile in the variation of the inflammatory markers. Given the correlations between inflammatory markers and the BMI percentile, we used an ROC curve analysis to evaluate the predictive power of BMI percentile, plasma glucose level and serum triglycerides on IL-6, ICAM and endotoxemia. The results indicated a significant predictive power of BMI percentile on inflammatory markers: IL-6 (AUC = 0.803, 95% CI: 0.72–0.88, *p* < 0.001), ICAM (AUC = 0.806, 95% CI: 0.72–0.89, *p* < 0.001) and endotoxemia (AUC = 0.762, 95% CI: 0.68–0.85, *p* = 0.019) (Table 3, Figure 2a,b). Plasma glucose level shows a significant prediction for IL-6 (AUC = 0.784, 95% CI: 0.63–0.93, *p* = 0.019) (Table 3, Figure 3a). Although a significant correlation with ICAM was observed in the case of serum triglycerides (*p* = 0.01), the results did not indicate a significant predictive power on any inflammatory marker (AUC = 0.60; 95% CI: 0.46–0.73, *p* = 0.129) (Table 3, Figure 3b).

### 3.3. Identifying Cut-Off Values for Inflammatory Markers in Obese Children and Adolescents

**Figure 4:**
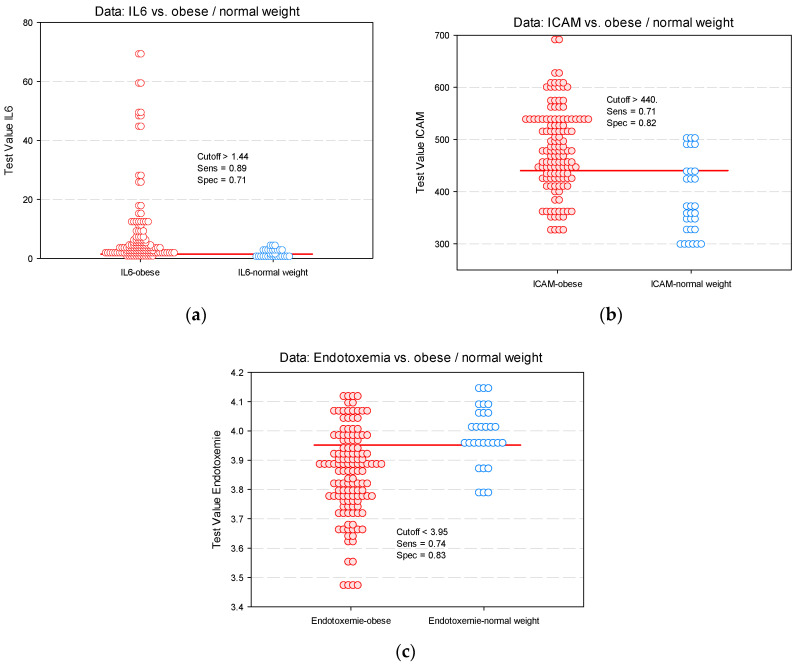
Identifying cut-off values predictive for vascular impairment in obese children (dot histogram) for: (**a**) IL-6; (**b**) ICAM; (**c**) endotoxemia.

Demonstrating the predictive power of BMI percentile and plasma glucose levels, we identified baseline cut-off values for IL-6, ICAM and endotoxemia for obese children and adolescents with early vascular damage (Figure 4).

### 3.4. Correlation of BMI Percentile and Biochemical Markers with Metabolic Markers for Insulin Resistance (Insulin, HOMA Index and Hormones Profile)

Evaluating the correlation between BMI percentile, metabolic markers and the analyzed biochemical parameters, we found that insulin value correlates significantly with BMI (*r* = 0.52, *p* = 0.001), total serum cholesterol (*r* = 0.265, *p* = 0.022) and triglycerides level (*r* = 0.228, *p* = 0.006) (Table 4).

The HOMA index correlates significantly with BMI (*r* = 0.516, *p* = 0.001), which is a significant predictive factor for the value of the HOMA index. Thus, the significant correlation between the HOMA index and the BMI percentile confirms that obesity is a major risk factor for the development of insulin resistance. The HOMA index also shows a significant correlation with total serum cholesterol (*r* = 0.273, *p* = 0.017) and with serum triglycerides (*r* = 0.205, *p* = 0.009) (Table 4). Plasma cortisol did not show significant correlations with total cholesterol, triglycerides or blood glucose levels and BMI (Table 4).

**Figure 5:**
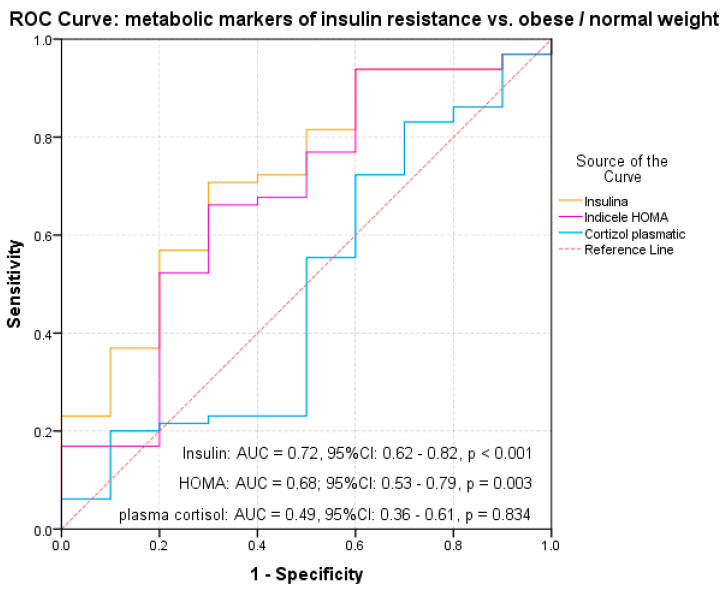
ROC curves for metabolic markers of insulin resistance and obesity in children and adolescents.

**Figure 6:**
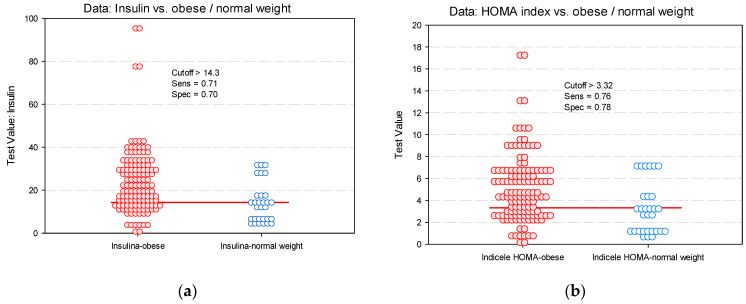
Identifying cut-off values predictive for vascular impairment in obese children and adolescents (dot histogram) for metabolic markers of insulin resistance: (**a**) insulin; (**b**) HOMA index.

Considering the results of the multivariate analysis, we could see that between BMI and the analyzed biochemical parameters, only the BMI percentile has a significant predictive power for metabolic markers of insulin resistance (insulin value: AUC = 0.72, *p* < 0.001 and HOMA index: AUC = 0.68, *p* = 0.003) (Table 5, Figure 5). This aspect was noted although the univariate analysis revealed other correlations between biochemical parameters (total cholesterol and triglycerides level) and insulin value or HOMA index. We noted that at insulin cut-off values of 14.3 (Figure 6a) and HOMA index cut-off of 3.32 (Figure 6b), respectively, the risk of vascular damage in obese children increases.

**Figure 7:**
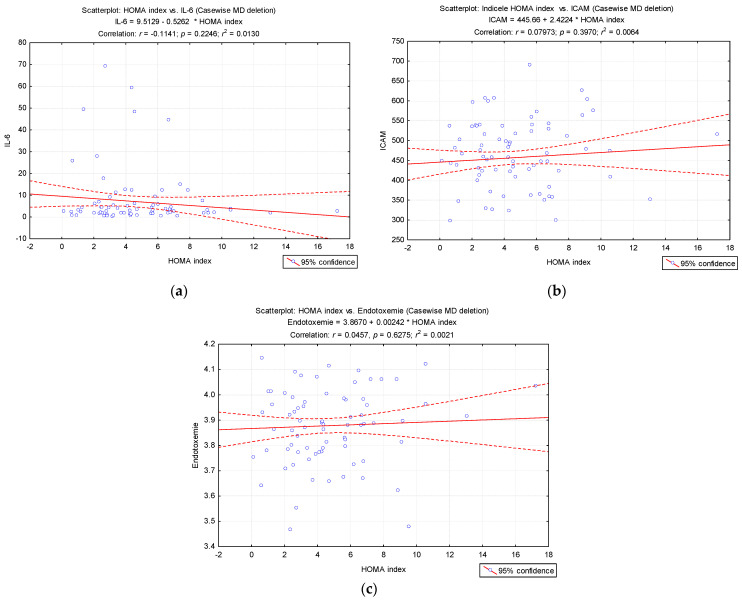
The regression line in the correlation of the HOMA index and (**a**) IL-6 values, (**b**) ICAM and (**c**) endotoxemia.

Because the evolution of obesity in our pediatric patients’ group is relatively short, we noticed no correlation between all these markers (HOMA index and IL-6, ICAM and endotoxemia) (Figure 7a–c). However, each individually assessed marker has a predictive value for the onset of cardiovascular and metabolic impairment in obese children.

## 4. Discussion

Identifying obese children at high risk for obesity in adulthood could be prevented by changing their lifestyle [15,16]. Chronic inflammation in the context of obesity is associated with the development of IR and atherosclerosis. Without significant interventions, the progression from obesity to IR and diabetes on the one hand, and to atherosclerosis, hypertension and CVD culminating in early mortality on the other hand, is inevitable [17,18]. Knowing and understanding the basic mechanisms associated with obesity-induced inflammation is necessary for therapeutic and even prophylactic programs, preventing irreparable complications. If it is intervened in time, in childhood and adolescence, it is possible to avoid or delay the appearance of these comorbidities [19,20].

One of the crucial aims to prevent pediatric obesity complications is to detect children with higher cardiovascular risk and IR at an early stage [14,21]. Among clinical markers, BMI percentile and WC are frequently used in practice but with certain limitations [22]. Although BMI is used as a measure of obesity, it is not sensitive enough to detect early fat accumulation and early CVD risk [14,23,24].

The role of low-grade inflammation as a link between obesity, IR and endothelial dysfunction is increasingly being discussed in the literature [25,26]. Low-grade inflammation was assessed in our study by measuring both IL-6 and ICAM. Thus, we observed that IL-6 was significantly correlated with blood glucose and BMI. The correlation between an elevated level of IL-6 and the occurrence of metabolic or cardiac comorbidities in adulthood has been demonstrated. Low-grade inflammation present in obese children is associated with changes in BP, especially systolic. It is known that BP exerts a pro-inflammatory effect on the arterial wall. IL-6 promotes the proliferation of vascular smooth muscle tissue, an early component of hypertension and atherosclerosis [4]. The presence of elevated values of IL-6 is associated with inflammation [27].

In our study, ICAM correlates significantly with serum triglycerides, blood glucose and BMI percentile; the BMI percentile has significant predictive power over ICAM. We did not find a positive correlation between the value of IL-6 and ICAM in the analyzed groups. A positive correlation was demonstrated between central obesity/insulin resistance and sICAM-1 levels, with sICAM-1 being considered a prototype inflammatory marker [2].

Along with soluble adhesion molecules such as E-selectin and vascular cell adhesion molecule 1 (VCAM-1), ICAM-1 represent a molecular marker of endothelial dysfunction. Increasing the levels of circulating adhesion molecules in obese patients plays an important role in the development of atherosclerosis [28]. sICAM-1 appears to reflect the extent of atherosclerotic lesions and is likely to be a predictive factor for future cardiovascular events in adulthood [4].

Researchers in Poland looked for the relationship between low-grade inflammation and high blood pressure in obese children and adolescents. They studied 281 overweight children aged between 6 and 18. Samples were collected to identify serum concentrations of C-reactive protein, IL-6, IL-1β, ICAM-1, VCAM-1, glucose and insulin. A significant correlation was found between CRP, IL-6, IL-1β and ICAM-1, with mean systolic blood pressure within 24 h. Inflammatory markers, CRP and IL-6 were correlated with mean diastolic BP at 24 h. The researchers found no relation between the ICAM-1 cell adhesion marker and the blood pressure of the patients examined but concluded that low-grade inflammation may play a role in modulating blood pressure relatively early in life [4].

Recently, researchers in Saudi Arabia studied markers of inflammation in obese children in the pre-pubertal period, trying to find a correlation between them and the metabolic syndrome. Weight, height, body mass index, systolic and diastolic pressure, blood glucose, C-reactive protein, IL-6 and sICAM-1 were analyzed in two groups of 25 obese and normal-weight children. Elevated levels of insulin, CRP, IL-6 and sICAM-1 have been observed in obese children. The glucose level was not shown to be altered in any of the groups. Furthermore, a correlation was found between sICAM-1 and the level of insulin, CRP and IL-6. The scientists concluded that obese patients in the pre-pubertal show changes that indicate insulin resistance, endothelial dysfunction and the presence of an inflammatory condition, and all this may increase the risk of developing cardiovascular disease and type 2 diabetes in adulthood [2].

In our study, endotoxemia has been significantly correlated with BMI. Metabolic endotoxemia is defined by the slight increase in plasma levels of endotoxins (lipopolysaccharides) from the intestine and having a pro-inflammatory role. The presence of lipopolysaccharides leads to increased lipid absorption and development of obesity, correlated with the value of BMI [29]. An increase in endotoxins of 0.5–2 times may be a good indicator for metabolic endotoxemia [30]. An intervention on the intestinal microbiota through diet and therapeutic measures may be important in reducing inflammation and endothelial dysfunction [28].

New studies look for a link between endotoxemia and obesity, diabetes and metabolic syndrome, as well as the link between the value of endotoxemia and other increased markers.

Researchers in Alexandria, Egypt studied endotoxemia in obese children and adolescents and its possible relationship with insulin, lipid profile and C-reactive protein. They studied 30 obese children and adolescents, aged between 5 and 18 years, and compared the results with those obtained from the control group, i.e., 20 patients with a normal weight. Lipid profile, liver function, endotoxin, C-reactive protein, glycemia and insulin resistance were analyzed. It was identified that endotoxin and CRP value were significantly higher in the obese group compared to the other group. The researchers also found that there is a positive correlation between serum endotoxin and body mass index, waist circumference, triglycerides, cholesterol and insulin. They concluded that endotoxin may play a role in cardio-metabolic risk factors associated with obesity in children and adolescents [31].

Another article published by Nigerian scientists demonstrates the link between patients with endotoxemia and obesity. They studied a number of 90 people divided into two groups, the first group consisting of 47 people with metabolic endotoxemia and the second of 43 people who formed the control group. Patients in the first group were further divided into three subgroups that included normal weight patients, the overweight group and the obese group. The researchers obtained positive relationships between the value of BMI and endotoxemia, between the amount of total cholesterol and endotoxemia, as well as between triglycerides and endotoxemia, all in patients in the first group. They concluded that the value of endotoxemia may precede the development of obesity [29].

In our study, insulin value was investigated among children and adolescents, trying to find a correlation with excess weight. We found that the values of insulin are correlated significantly with TG and BMI percentile, but only the BMI percentile has predictive power over insulin.

Scientists in Germany have identified possible associations between the value of insulin, glycemic index and blood sugar during puberty in children with body composition in adulthood. The study was based on the hypothesis that regular consumption of a diet in young people induces higher levels of postprandial blood sugar, and that high amount of insulin, can have an unfavorable effect on the composition of the body in adulthood. The study was performed on 262 people in whom two dietary recordings were made once every 3 days during puberty and the evaluation of anthropometric measurements at the age of 18–25 years. No correlation was found between glycemic index or glycemic load at puberty with body composition in adulthood, but it was observed that a high insulin value during puberty was associated with a higher percentage of body fat in adulthood. Thus, it was concluded that the postprandial increase of insulinemia, from the pubertal period, plays a role in the defective development of the body composition in adulthood [32].

Researchers in the United States conducted a study to identify a correlation between serum insulin levels and insulin resistance on body fat growth in African-American and Caucasian children. They studied 249 children aged between 6 and 12 years, healthy but at high risk of obesity in adulthood due to overweight in childhood or due to overweight parents. Subjects were followed until the age of 15, during which time anthropometric and biochemical measurements were performed periodically. At the start of the study, 39% of children had a BMI percentile above the 95th percentile. No correlation was found between insulin value, glycemic index, fat percentage or BMI percentile, concluding that during childhood, BMI value and amount of adipose tissue play an important role in the further development of an obese adult. They did not find a correlation between plasma insulin levels and obesity, suggesting that this relation needs to be studied [33].

The HOMA index has significantly correlations only with BMI percentile, which is a significant predictive factor for the value of the HOMA index. This correlation confirms that obesity is a major risk factor for the development of insulin resistance. This aspect was also validated in the study of Elnashar et al. [2], who showed that insulin and the HOMA index have a significant increase in obese children. Positive correlations were fund between insulin and HOMA and BMI percentile, systolic BP, LDL cholesterol and TG as metabolic parameters and between insulin and HOMA and CRP, IL-6 and ICAM 1 as an inflammatory parameter. According to this, in our study, we found a significant correlation between cardiovascular risk factors and insulin resistance defined by HOMA. Some studies mention that it is necessary to take into account the differences between sexes in the pathophysiology of obesity [34].

Starting from the obtained results, we want to carry out in the future a complex analysis regarding the changes of inflammation and insulin resistance markers that appear in the case of cardiovascular complications in obese children and adolescents. We will use a clustered form of analysis that will significantly increase the power of statistical prediction estimates [35].

The tests performed in our study did not identify significant correlations between the value of plasma cortisol and the levels of cholesterol, TG and glycemia, nor with BMI percentile, a fact confirmed by Abraham′s study [9], who did not find any correlation between BMI percentile or weight and cortisol (either salivary or urinary/24 h), and no correlation between cortisol and the values of TG, HDL and BP.

The limitations of our study were: the relatively small number of the patients in the both groups and the impossibility of evaluating fat mass (FM) and fat free mass (FFM) through densitometry or dual-energy X-ray absorptiometry. Although BMI is widely used as a surrogate measure of adiposity, it is a measure of excess weight, rather than excess body fat [36]. We will evaluate in future studies the parameters related to the composition of body mass.

However, in a meta-analysis of four studies, overweight and obese children who became normal in adulthood were no different in terms of more cardiovascular disease risk parameters than those patients who were never obese [37]. Thus, early nutritional interventions instituted in obese children that lead to weight loss will contribute to the health of future adults.

## 5. Conclusions

An adequate understanding of the inflammatory processes characteristic of obesity constitutes a crucial factor for the prevention of the disease and its complications in obese pediatric patients.

The study highlighted the importance of early markers of cardiovascular risk in obese pediatric patients represented by interleukin 6 (IL-6), Intercellular Adhesion Molecules (ICAM), endotoxemia and their correlation with metabolic markers of insulin resistance (IR) represented by insulinemia, HOMA index and plasma cortisol.

Thus, inflammatory markers, IL-6, ICAM 1 and endotoxemia, show significantly higher values in pediatric obese patients, leading to chronic and systemic inflammation. The results of the study indicated that these markers can be considered significant predictors of cardiometabolic diseases in these patients.

Of particular interest is the link between low-grade inflammation, IR and endothelial dysfunction and obesity. The significant correlation between the HOMA index and the BMI percentile confirms that obesity is a major risk factor for the development of insulin resistance.

It can clearly be considered that the BMI percentile has significant predictive power for metabolic markers of insulin resistance. However, our study highlights the need for detailed research in the dynamics of obese pediatric patients by age group.

## Figures and Tables

**Table 1:** Comparison of clinical and biochemical parameters between the group of obese pediatric patients and the control group.

Baseline Characteristics	Study Group (*n* = 115)				Statistical Test	*p*-Value
	Control Group (*n* = 30)		Obese Pediatric Patients (*n* = 85)			
	Mean ± SD	Std.Err.	Mean ± SD	Std.Err.		
Age: Years ^†^	**13.4 ± 2.47**	0.34	12.1 ± 3.4	0.29	2.40	0.142
Gender, (boys/girls) ^‡^	12/18 (40%/60%)		44/41 (51.8%/48.2%)		1.24	0.267
BMI (kg/m2) ^†^	19.2 ± 1.8	0.28	29.2 ± 5.2	0.47	27.64	<0.001 *
Percentiles BMI ^†^						
mean ± SD	40.6 ± 2.3	0.75	97.52 ± 2.4	0.21	65.16	<0.001 *
median (Q1; Q3)	40.5 (17; 58)		98 (97; 99)			
Percentiles Waist Circumference ^†^						
mean ± SD	42.8 ± 4.6	0.50	93.9 ± 4.6	0.41	35.78	<0.001 *
median (Q1; Q3)	44.5 (34; 50)		95 (92; 97)			
Lipid profile and liver function tests ^†^						
Total serum cholesterol (mg/dL)	152.3 ± 14.1	2.57	165.7 ± 30.7	2.63	5.29	0.023 *
Triglycerides (mg/dL)	44.1 ± 5.6	1.02	104.1 ± 51.96	4.33	39.67	<0.001 *
LDL cholesterol	64. 3 ± 5.5	0.98	79.9 ± 24.4	2.03	12.16	0.0006 *
HDL cholesterol	79.2 ± 11.7	1.65	64.9 ± 15.2	1.34	22.03	<0.001 *
ALT	13.6 ± 8.7	1.58	24.1 ± 15.9	1.39	11.58	<0.001 *
AST	18.7 ± 7.2	1.31	22.4 ± 8.9	0.78	4.34	0.039 *
TP	70.8 ± 4.1	0.62	73.3 ± 3.4	0.30	10.27	0.001 *
Glucidic profile and insulin resistance ^†^						
Plasma glucose level	85.3 ± 9.5	1.03	89.3 ± 11.2	1.82	0.65	0.438
Hb A1c	4.1 ± 0.4	0.07	4.8 ± 0.5	0.04	14.06	0.0002 *
Insulin, μU/mL	13.8 ± 9.2	1.57	23.3 ± 14.8	1.36	10.95	0.001
HOMA index	3.17 ± 2.3	0.32	4.94 ± 2.9	0.21	8.85	0.003 *
Inflammatory markers and the hormones profile ^†^						
IL6	1.68 ± 1.3	0.22	9.08 ± 15.1	1.25	7.14	0.008 *
ICAM 1	385.6 ± 71.7	8.09	481.6 ± 79.7	6.94	33.80	<0.001 *
Endotoxemia	3.98 ± 0.1	0.01	3.83 ± 0.2	0.01	24.08	<0.001 *
Plasma cortisol	184.7 ± 108.4	9.78	176.1 ± 105.9	9.63	0.145	0.704
Blood pressure ^†^						
SBP, mm Hg	102.3 ± 5.2	0.94	117.7 ± 14.1	1.22	34.13	<0.001 *
DBP, mm Hg	60.7 ± 1.6	0.30	73.7 ± 12.5	1.07	32.3	<0.001 *
Blood pressure value ^‡^					20.83	0.0003 *
Normal	30 (100%)		46 (54.1%)			
Borderline hypertension	0 (0%)		16 (18.8%)			
Hypertension	0 (0%)		23 (27.1%)			

Continuous variables were expressed as: mean ± standard deviation; Std.Err.—standard error; categorical variables: number (%). Q1; Q3—lower quartile; upper quartile. ^†^ Student’s *t*-test or Mann-Whitney U Test for continuous variables. ^‡^ Pearson Chi-square test. * Marked effects are significant at *p* < 0.05.

**Table 2:** Univariate analysis showing correlations between inflammatory markers and biochemical parameters and BMI percentile.

Dependent Variable	Independent Variable	Correlation Coefficient (Pearson Correlations)	*p*-Value
IL6 vs.	Total serum cholesterol	−0.039	0.318
	LDL-cholesterol	−0.0633	0.427
	Triglycerides	−0.034	0.341
	Plasma glucose level	−0.334	0.001 *
	BMI percentile	0.252	0.031 *
ICAM vs.	Total serum cholesterol	0.121	0.072
	LDL-cholesterol	0.208	0.008 *
	Triglycerides	0.253	0.001 *
	Plasma glucose level	−0.145	0.044 *
	BMI percentile	0.302	0.037 *
Endotoxemia vs.	Total serum cholesterol	0.082	0.166
	LDL-cholesterol	−0754	0.343
	Triglycerides	−0.035	0.335
	Plasma glucose level	−0.346	0.042 *
	BMI percentile	−0.255	0.001 *

* Marked effects are significant at *p* < 0.05.

**Table 3:** The coefficients of multiple linear regression regarding the correlations between early inflammatory markers of cardiovascular risk and BMI percentile and biochemical parameters.

Multiple Linear Regression	Unstandardized Coefficients		Standardized Coefficients	t-Value	*p*-Value
	B	Std. Error	Beta		
**Dependent Variable: IL-6**					
Total serum cholesterol	0.026	0.042	0.054	0.607	0.545
LDL-cholesterol	−0.126	0.016	−0.207	−1.192	0.235
Triglycerides	−0.050	0.026	−0.184	−1.898	0.060
Plasma glucose level	−0.413	0.091	−0.254	−3.520	0.024 *
BMI percentile	0.631	0.054	0.421	2.411	0.017 *
Model verification: ANOVA, F = 4.815, *p* = 0.001 *					
**Dependent Variable: ICAM**					
Total serum cholesterol	0.095	0.168	0.032	0.355	0.723
LDL-cholesterol	1.053	0.154	0.267	1.610	0.109
Triglycerides	0.429	0.064	0.250	2.621	0.010 *
Plasma glucose level	−0.726	0.177	−0.100	−1.257	0.211
BMI percentile	0.787	0.142	0.244	2.883	0.005 *
Model verification: ANOVA, F = 4.098, *p* = 0.003 *					
**Dependent Variable: Endotoxemia**					
Total serum cholesterol	0.001	0.001	0.110	1.347	0.180
LDL-cholesterol	0.021	0.001	−0.012	−0.149	0.881
Triglycerides	−0.002	0.001	−0.014	−1.174	0.083
Plasma glucose level	−0.032	0.001	−0.013	−0.195	0.694
BMI percentile	0.452	0.001	0.276	3.185	0.038 *
Model verification: ANOVA, F = 5.873, *p* = 0.028 *					

* Marked effects are significant at *p* < 0.05.

**Table 4:** Univariate analysis showing correlations between metabolic markers of insulin resistance and biochemical parameters and BMI percentile.

Dependent Variable	Independent Variable	Correlation Coefficient (Pearson Correlations)	*p*-Value
Insulin value vs.	Total serum cholesterol	0.265	0.022 *
	Triglycerides	0.228	0.006 *
	Plasma glucose level	−0.126	0.142
	BMI percentile	0.522	0.001 *
HOMA index vs.	Total serum cholesterol	0.273	0.017 *
	Triglycerides	0.205	0.009 *
	Plasma glucose level	0.132	0.092
	BMI percentile	0.516	0.001 *
Plasma cortisol vs.	Total serum cholesterol	0.037	0.326
	Triglycerides	0.027	0.372
	Plasma glucose level	0.042	0.596
	BMI percentile	0.144	0.067

* Marked effects are significant at *p* < 0.05.

**Table 5:** The coefficients of multiple linear regression regarding the correlations between metabolic markers of insulin resistance and BMI percentile and biochemical parameters.

Multiple Linear Regression	Unstandardized Coefficients		Standardized Coefficients	t-Value	*p*-Value
	B	Std. Error	Beta		
**Dependent Variable: Insulin Value**					
Total serum cholesterol	0.061	0.049	0.115	1.231	0.220
Triglycerides	−0.018	0.030	−0.060	0.603	0.548
Plasma glucose level	−0.105	0.106	−0.081	−0.992	0.323
BMI percentile	1.217	0.063	0.512	6.820	<0.001 *
Model verification: ANOVA, F = 14.712, *p* < 0.001 *					
**Dependent Variable: HOMA Index**					
Total serum cholesterol	0.011	0.010	0.101	1.085	0.280
Triglycerides	−0.004	0.006	−0.072	−0.718	0.474
Plasma glucose level	0.031	0.021	0.119	1.475	0.142
BMI percentile	0.430	0.013	0.520	7.019	<0.001 *
Model verification: ANOVA, F = 16.186, *p* < 0.001 *					
**Dependent Variable: Plasma Cortisol**					
Total serum cholesterol	0.111	0.370	0.029	0.301	0.764
Triglycerides	−0.013	0.228	−0.006	−0.441	0.660
Plasma glucose level	0.339	0.796	0.036	0.425	0.571
BMI percentile	2.767	0.472	0.060	0.668	0.062
Model verification: ANOVA, F = 4.815, *p* = 0.001 *					

* Marked effects are significant at *p* < 0.05.

## Data Availability

The data presented in this study are available on request from the corresponding author.
